# Unlocking the Potential Use of Berry Anthocyanins as Pharmaceutical Excipients and Nanocarriers: Evidence from the Last Decades

**DOI:** 10.3390/ijms27062562

**Published:** 2026-03-11

**Authors:** Ana C. Gonçalves, Maria de São-José Matias, Rafael Fonseca, Luís R. Silva

**Affiliations:** 1 RISE-HEALTH, Health Sciences Research Center, University of Beira Interior, Av. Infante D. Henrique, 6200-506 Covilhã, Portugal; 2 Faculdade de Ciências da Saúde, Universidade da Beira Interior (FCS-UBI), 6200-506 Covilhã, Portugal; maria.sj.matias@ubi.pt; 3 Faculty of Medicine, University of Lisbon, 1649-028 Lisbon, Portugal; rafaelfonseca1@edu.ulisboa.pt; 4 CERES, Department of Chemical Engineering, University of Coimbra, 3030-790 Coimbra, Portugal; 5 SPRINT Sport Physical Activity and Health Research & Innovation Center, Instituto Politécnico da Guarda, 6300-559 Guarda, Portugal

**Keywords:** biological potential, natural products, pharmaceutical excipients, nanocarriers, anthocyanins

## Abstract

Secondary metabolites, particularly natural phenolic compounds, have been a target of many studies and are a hot issue in the medical and scientific communities, due to their diverse biological activities, including antioxidant, anti-inflammatory, and antimicrobial effects. This bioactive potential has raised the prospect of their application as pharmaceutical excipients and nanocarriers. Among them, anthocyanins, which are abundant in berries and highly valued by consumers, stand out as promising candidates. Their chemical structure not only enables them to protect drugs from oxidative degradation but also supports their role in drug delivery systems, particularly under acidic conditions. Moreover, their pH-dependent color changes make them suitable as eco-friendly indicators and sensors. The current review aims to summarize recent advances on the excipient and nanocarrier potential of berry phenolics. Although current data on anthocyanins as excipients and nanocarriers remain limited, available evidence highlights their potential and urges additional in vitro, in vivo, and clinical studies.

## 1. Introduction

Currently, there is an increment in the search for natural elements to be used in several areas worldwide, including in food, cosmetic, and pharmaceutical industries [1,2]. Among the various alternatives, phenolic compounds are a hot topic of discussion and search around scientific and medical communities, appearing to be a useful strategy since they present several applications [1,3,4]. In reality, they are widely distributed in nature, are easy to obtain, inexpensive and, unlike certain pharmaceuticals, have few, if any, adverse effects, making them regarded as harmless and non-toxic to human health [5]. In addition, they exhibit several health-promoting properties, including the capability of normalizing inflammatory cytokines and oxidative stress to basal levels, attenuating, or even preventing, the development of many disorders, like cancer, metabolic syndrome, and hepatic, neurological, and cardiovascular pathologies, and, of course, boosting the immune system [6,7,8,9].

Focusing on pharmaceutical excipients and nanocarriers, data suggests that their market would rise by roughly 6% in 2033 compared with 2024, reaching around USD 14.72 billion in ten years, taking an increasingly important position in the market [10]. Despite this, they are responsible for about 45% of allergy and immune reactions, increasing angioedema, bronchoconstriction, hyperactivity, skin eruption events, and gastrointestinal symptoms, among others [11,12].

An excipient is considered “substances other than the active pharmaceutical ingredient that have been appropriately evaluated for safety and are intentionally included in a drug delivery system” [13]. On the other hand, a nanocarrier is an “advanced form of excipient, supporting drug formulation and delivery, and also offers additional health-related benefits, such as better solubility and stability, targeted or controlled drug release, protection of the active ingredient, and in some cases, intrinsic therapeutic effects” [14].

Both can be classified according to their (i) route of administration (oral, topical, parenteral or other), or (ii) origin [inorganic (e.g., halites, metallic oxides, or calcium carbonate, phosphate or sulfate) or organic chemicals (e.g., acrylic polymers, carbohydrates, cellulose, glycols, mineral hydrocarbons, oleochemicals, proteins, starch, among others)] [15]. In the development of each formulation, the main roles of excipients and nanocarriers include protection, support, and enhancing stability and bioavailability of the formulation, aiding in the processing of the drug delivery system, and contributing to the effectiveness, delivery, and maintenance of the integrity of the drug, as well as to its overall safety and patient acceptability [16,17]. The ideal ones need to be reproducible and stable, cost-effective, pharmacologically inert, and present desired functionality with no ability to cause unwanted drug interactions [15,18].

Phenolic compounds extracted from natural products seem to be a useful approach. The main goal of this work is to provide a detailed review of the potential of berry phenolics to be pharmaceutical excipients and nanocarriers, discussing the more recent research. This overview was divided into three sections. The first two sections describe the main sources of phenolics in nature and their industrial use. The last section summarizes the latest findings and discusses the excipient and nanocarrier potential of berry phenolics, focusing on evidence from the last ten years. Berry anthocyanins are chosen because they are present at considerable levels in berries and other red fruits and vegetables; in addition, industrial applications and biological potential are a focus of many studies and consumer acceptance. Furthermore, as expected, berry production is increasing around the world, with an increase in their production up 42% in the last ten years [19].

## 2. Data Collection

The process of gathering data involved searching in Google Scholar, National Center for Biotechnology Information, ResearchGate, PubMed, Science Direct, Scopus, and SpringerLink up to March 2025. The free terms, MeSH terms, and keywords applied were anthocyanins, natural phenolic sources, polyphenols, phenolics, nanoencapsulation, pharmaceutical excipient, and pharmaceutical nanocarrier, paired with AND, OR, or NOT operators. During the literature review, there were no limitations on the author(s) or publication type. Only articles written in English and published in scientific journals were considered. In total, 112 papers were cited in the present overview.

## 3. Main Sources of Anthocyanins

**Figure 1:**
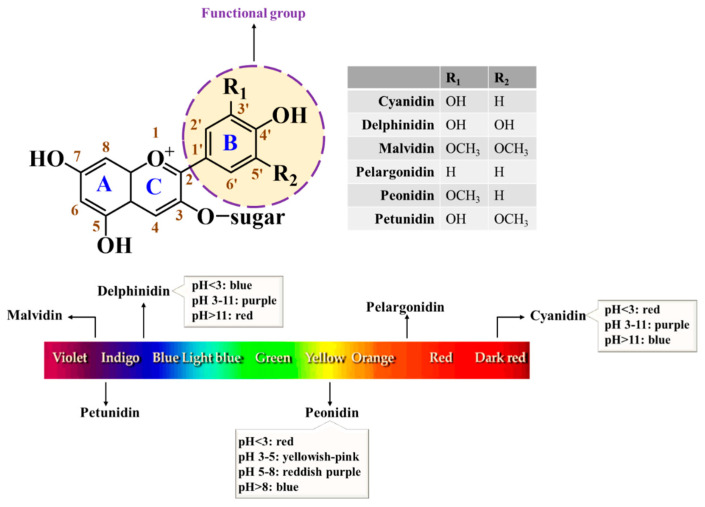
Chemical structure of the most predominant anthocyanins in nature, and their color in accordance with pH values (adapted from Câmara et al. [2] and Gonçalves et al. [49]).

From a botanical point of view, berries are defined as “corpulent fruits in which the ovary of a single flower develops into an edible fleshy portion, i.e., the pericarp” [20]. Most berries are small, edible, seasonal, and perishable fruits that present a round shape, attractive colors, and pleasant aroma and flavor, being largely appreciated by consumers [21]. In addition, their richness in phenolic compounds, namely anthocyanins, confers them notable antioxidant effects and the capacity to chelate metals and interfere with inflammatory pathways, contributing to increasing their interest and, of course, to a healthy status [22,23]. Anthocyanins (anthocyanidin glycosides) are mainly responsible for the color and health-promoting properties shown by these fruits, mainly thanks to their catechol, pyrogallol, and methoxy groups [24], as mentioned in Figure 1. As far as we know, anthocyanins are more stable at low pH values, and their biological potential is greatly influenced by sugar and number of moieties units attached to the aglycone, as well as by the methylation degree, number and position of the hydroxyl groups, as well as by the position of aromatic and/or carboxylated aliphatic acids on the sugar residue [25,26,27]. Hydroxyl groups (OH) in phenolic compounds are generally considered important contributors to antioxidant activity due to their ability to donate hydrogen atoms and stabilize free radicals [28]. Nevertheless, the relationship between the number of hydroxyl groups and antioxidant capacity is not strictly linear. Antioxidant activity is influenced by several structural factors, including the position and orientation of hydroxyl groups, the degree of conjugation within the molecule, glycosylation patterns, and interactions with other constituents. In this context, structural differences among anthocyanidins, such as the degree of hydroxylation and methoxylation, may partly explain variations in antioxidant activity. For example, the higher hydroxylation of delphinidin compared with petunidin may contribute to its greater antioxidant potential [26,28,29].

The most consumed berries around the world include Açaí, bananas, bearberries, bilberries, black mulberries, blackberries, blackcurrants, blueberries, boysenberries, chokeberries, cranberries, cloudberries, elderberries, goji, gooseberries, grapes, huckleberries, lingonberries, raspberries, strawberries, and tomatoes. Table 1 summarizes the total phenolic and anthocyanin content of them. Their levels depend on several factors, like genotype, origin, climate, agricultural processing and storage practices, and time of harvesting [30,31,32,33].

In a general way, cyanidin 3-*O*-glucoside and cyanidin 3-*O*-rutinoside are the major anthocyanins found in açaí samples, with total concentrations in the range between 3.6 and 14.3 cyanidin 3-glucoside equivalents mg/g [34]. Cyanidin 3-*O*-rutinoside (60%) is also the most abundant anthocyanin found in black mulberries, followed by cyanidin 3-*O*-glucoside (38%) [35]. On the other hand, cyanidin 3-*O*-galactoside, cyanidin 3-*O*-arabinoside, and cyanidin 3-*O*-glucoside are the main reported compounds in bearberry fruits with amounts of 127, 2.79, and 0.88 mg per 100 g fw [36]. Cyanidin 3-*O*-galactoside was also the major anthocyanin found in bilberries, representing 43% of the total peak area [37], and also in lingonberries, with percentages varying between 74.4% and 83.5% of total anthocyanins [30], and in chokeberries (c.a. values of 229 mg per 100 g fw) [36]. In blackberries, the highest levels of cyanidin 3-*O*-glucoside were found, accounting for between 43.6 and 95.2% of total anthocyanins [38,39]. In relation to blackcurrants, the most represented anthocyanin is delphinidin 3-*O*-rutinoside (36.7–63.6%), followed by cyanidin 3-*O*-rutinoside (26.4–40.6%), delphinidin 3-*O*-glucoside (6.1–17.9%), and cyanidin 3-*O*-glucoside (1.3–9.9%) [31]. Delphinidin 3-*O*-rutinoside is also the major anthocyanin found in gooseberries (60.4% of total anthocyanins), followed by cyanidin 3-*O*-rutinoside (20.9% of total anthocyanins) [40].

Blueberries present considerable levels of delphinidin 3-*O*-galactoside, malvidin 3-*O*-galactoside, malvidin 3-*O*-arabinoside, cyanidin 3-*O*-arabinoside, and delphinidin 3-*O*-arabinoside; together, they constituted about 70% of total anthocyanins [41,42]. Concerning boysenberries, cyanidin 3-*O*-sophoroside, cyanidin 3-glucosylrutinoside, cyanidin 3-*O*-glucoside, and cyanidin 3-*O*-rutinoside are the most representative anthocyanins in this berry [39]. Cyanidin 3-*O*-sophoroside is also the most representative anthocyanin in cloudberry fruits, followed by cyanidin 3-*O*-glucoside, at levels of 0.86 and 0.83 mg per 100 g fw, respectively [36]. Raspberries also present considerable amounts of cyanidin-3-sophoroside, as well as cyanidin-3-(2G-glucosylrutinoside) [43]. Elderberries possess considerable amounts of cyanidin 3-p-coumaroyl-sambubioside 5-glucoside, cyanidin 3-sambubioside 5-glucoside, and cyanidin 3-sambubioside (151.0, 46.4, and 63.9 mg per 100 g fw, respectively) [36].

Cyanidin 3-*O*-sambubioside is the most represented anthocyanin in cranberry fruits (26.7 mg per 100 g fw) [36], while petunidin 3-*O*-rutinoside(trans-p-coumaroyl)-5-*O*-glucoside is the major anthocyanin present in goji berries, representing more than 80% of total anthocyanin content [44]. In huckleberries, cyanidin 3-*O*-arabinoside, cyanidin 3-*O*-glucoside, and cyanidin 3-*O*-galactoside are the most common (2.71–111.02, 8.84–42.36, and 0.77–14.81 mg per 100 g fw, respectively) [45]. Malvidin 3-*O*-glucoside was the most representative anthocyanin in grapes (49%), followed by malvidin 3-(6″-*O*-coumaroyl)glucoside (12%) and peonidin 3-*O*-glucoside (10%) [46].

The two major anthocyanins reported in strawberry fruits are pelargonidin 3-*O*-glucoside (89–95% of total anthocyanin content) and cyanidin 3-*O*-glucoside (3.9–10.6%) [22,47]. Regarding tomato, the most represented phenolics in them are non-coloured compounds, namely chlorogenic acid (3.7 mg/100 g fw) and rutin (5 mg/100 g fw); petunidin-3-(trans-p-coumaroyl)-rutinoside-5-glucoside and malvidin-3-(trans-p-coumaroyl)-rutinoside-5-glucoside (accounting 56.6% and 21.4% of total anthocyanins, respectively) are the most common anthocyanins [48].

**Table 1:** Main berries’ phenolic-rich sources.

Berries	TPC (mg GAE per 100 g of fw)	TA (mg per C3G per 100 g of fw)	References
Açaí	6.1–517.8	0.57	[50,51]
Bearberries	527.0	133.0	[36]
Bilberries	758.6	329.0	[37]
Black mulberries	195.1–273.3	50.8–71.2	[50]
Blackcurrants	7.8–380.0	1.82–231.8	[23,31,51]
Blueberries	275.0–1974.8	28.55–322.5	[52,53]
Blackberries	336.3–502.8	103.5–271.8	[32,54]
Boysenberries	39.2–357	1.03–146.2	[54,55]
Chokeberries	603.0	357.0	[36]
Cranberries	762.0	29.6	[36]
Cloudberries	71.7–280.93	2.22	[36]
Elderberries	356.0	211.0	[36]
Goji	162.4–901.0	196.0	[56,57]
Gooseberries	197.0–5950.0	2.5.0–280.9	[58,59,60]
Grapes	13.8–149.0	50.0	[61,62]
Huckleberries	281.0–284.0	11.0–31.0	[63]
Lingonberries	468.0–661.0	20.0–57.0	[30,64]
Raspberries	196.6–391.2	1.3–321.0	[58,60,65]
Strawberries	190.0–570.0	38.0–190.0	[66]
Tomato	18.6–55.9	7.1.0	[33,48]

GAE: gallic acid equivalents; C3G: cyanidin 3-*O*-glucoside equivalents; fw: fresh weight; TPC: total phenolic content; TA: total anthocyanins.

## 4. Emerging Applications of Anthocyanins in Food, Packaging, and Nutraceutical Fields

**Figure 2:**
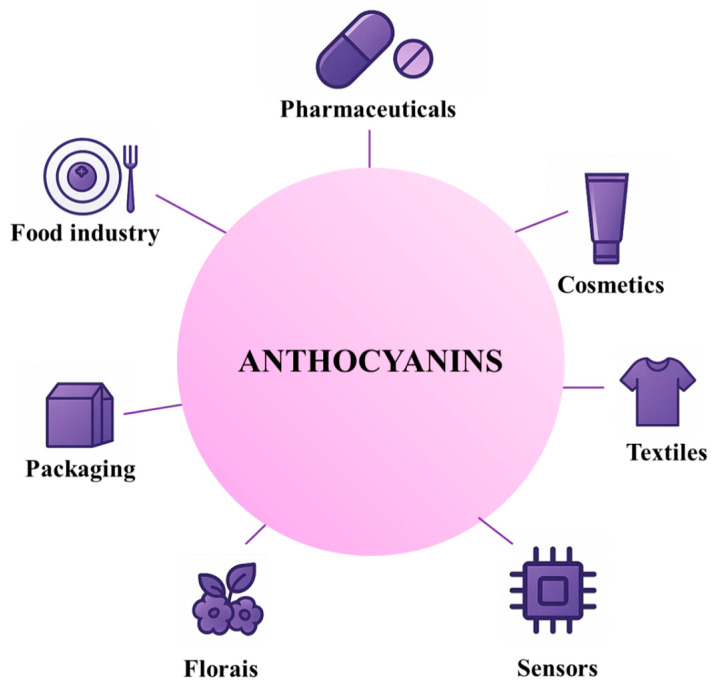
General industrial applications of anthocyanin compounds (adapted from Gonçalves et al. [18]).

Industries, especially food and beverage industries, use many synthetic substances as colorants; however, their continuous utilization is unhealthy and presents several side effects, like allergic reactions, migraine headaches, cancer, and hyperactivity in sensitive children [66,67]. In order to solve these contraindications, many efforts have been made, and currently, anthocyanins are considered a good alternative, being safer and healthier [68]. In fact, their use as an alternative to synthetic ones has already been approved in the European Union (E 163), Japan, and the United States of America [68,69]. They can be added to bakery, pastry, and dairy products, jams and jellies, soft drinks, syrups, and powders, and in addition to providing color to the foods and beverages, they also protect them against damage and increase their nutritional potential and health benefits for consumers (Figure 2).

Focusing on the food industry, and as already mentioned, anthocyanins, Rodríguez-Mena et al. [70] already revealed that adding these colored compounds extracted from purple sweet potatoes to ice pops can maintain their color and soluble compounds and stabilize their storage pH for 21 days. Similar results have been obtained by Albuquerque and colleagues [71], who related that the incorporation of jaboticaba epicarp anthocyanins in macarons stabilizes their color for more than 6 days when compared with synthetic colorants. Another work, conducted by Lopez and colleagues [72], showed that cyanidin 3-*O*-glucoside extract extracted from *Arbutus unedo* L. and incorporated into wafers makes them more attractive and increases their antioxidant abilities. Finally, the incorporation of anthocyanin-rich extracts into gummy candies can enhance their colorimetric sensory qualities [73]. Altogether, and in accordance with evidence, the better sources of anthocyanins to be used as food colorants include red and purple fruits and vegetables, grape skin, black beans, black carrots, and *Hibiscus sabdariffa* flowers [67,74].

It has also been reported that the addition of anthocyanins from bokbunja fruits to jams at pH values of 2–3, rather than those above 3.5, provides added value by maintaining higher levels of phenolics [75]. Furthermore, Shamshad et al. [76] reported that the application of anthocyanins (143.2 ± 1.1 mg per 100 g) from black carrots to ice cream can enhance its quality and sensory attributes. Similar results were reported by Aguilar et al. [77], who reported that the addition of anthocyanins extracted from grapes can enhance the nutritional value of beverages. Furthermore, the addition of grape anthocyanins into apple puree enhances twofold higher antioxidant potential and improves antiglycation properties compared with plain apple puree [78].

Regarding food packaging, Zhai et al. [79] reported that colorimetric films composed of roselle anthocyanins and starch/polyvinyl alcohol enhance fish quality by reducing their water content. In addition, they also refer that anthocyanins exhibit significant antioxidant and antimicrobial properties in edible films. Still regarding fish preservation, Silva-Pereira et al. [80] reported that chitosan/corn starch blend films with extract from red cabbage can be used as a visual indicator of fish deterioration since they are very sensitive to pH changes. In addition, Sani and colleagues [81] reported that the addition of red barberry anthocyanins in carbohydrate-based films enhances their good antioxidant and antimicrobial activity. Similar data was obtained by Wang and colleagues [82], who reported that the addition of cranberry anthocyanins on chitosan hydrochloride and carboxymethyl chitosan films improves their mechanical properties, thermal stability, and antioxidant capacity, and also reduce the oxidative degradation of olive oil, showing a peroxide value of 21.2 meq O_2_ per kg after 56 days of storage, being more efficient than films composed of gelatine only, which exhibited a peroxide value of 28.4 meq O_2_ per kg after 56 days of storage. Finally, the mixture of saffron petal anthocyanins with chitosan nanofiber/methyl cellulose matrices increases tensile strength, light-screening properties, antimicrobial activity against *Escherichia coli* and *Staphylococcus aureus*, and antioxidant capability against 2,2-diphenyl-1-picrylhydrazyl radicals of the films [83].

Furthermore, it is important to note that several reports highlight the potential of anthocyanins to be nutraceuticals. For example, Cassidy and colleagues demonstrated that regular consumption of anthocyanins and flavanones is positively associated with nonfatal myocardial infarction [84]. Their study involved 93,600 healthy women, who daily ingest 58–643 mg per day of flavonoids and 2–35 mg per day of anthocyanins for 4 years. In another study, these authors also reported that the same diet is effective in contributing to the prevention of hypertension [85]. In a prospective study conducted by Yang and Lee reported that a higher intake of anthocyanins can also improve hyperlipidemia status [86]. Moreover, it has also been reported that the dietary intake of polyphenols (2543 mg per day), including anthocyanins, increases short-term, long-term memory and lexical-semantic memory, and learning in postpartum Argentinian women [87]. In addition, Mulleder et al. [88] explored the effects of simultaneous consumption of anthocyanins (mainly cyanidin 3-*O*-glucoside and cyanidin 3-*O*-sambubioside) from various red fruits, including blackcurrant, blueberries, red grape, and elderberry, with sucrose and found that sucrose promoted the bioavailability of anthocyanins in humans. This data reinforces the possibility that the absorption and assimilation in the human body is influenced by the presence of other compounds, e.g., sugars [89].

Even so, it is also important not to forget that sometimes their inclusion alters products’ flavor, particularly when anthocyanins are extracted from red radish and beet [74]. In addition, their use and application are not as easy as they seem. Firstly, they are more expensive than synthetic dyes, given that the application of efficient extraction techniques is necessary [74]. Moreover, as mentioned before, anthocyanins’ stability and color are largely influenced by the presence of oxidants, enzymes, and metals, temperature, cooking processes, oxygen, pH, and light [67,90]. The acylated anthocyanins are usually preferred to be used as food colorants because of their higher stability [68]. Besides, it is also important to know that lower pH values contribute to purple, red, and blue colors, while in basic pH values, anthocyanins become unstable, and although they turn to dark brown in the first stage, they end up degrading and becoming uncolored over time [67,90,91]. To circumvent and avoid their degradation, several experiences have been performed, standing out those that promote acylation processes and oxygen exclusion, freeze and spray-drying [67]. Microencapsulation and nanoemulsion techniques are also targets of many studies [92]. Indeed, Carmona and colleagues [93] reported that maltodextrin and mucilage-maltodextrin enriched with anthocyanins extracted from cactus pear can retain the color, pigment, and quality of yogurts for 28 days of storage. In addition, Ab Rashid et al. [94] used *Clitoria ternatea* flower anthocyanins to produce more durable maltodextrin microcapsules to be exposed to light for up to 21 days and to act against foodborne bacteria on muffins.

## 5. Exploring Anthocyanins Use as Pharmaceutical Excipients and Nanocarriers

**Figure 3:**
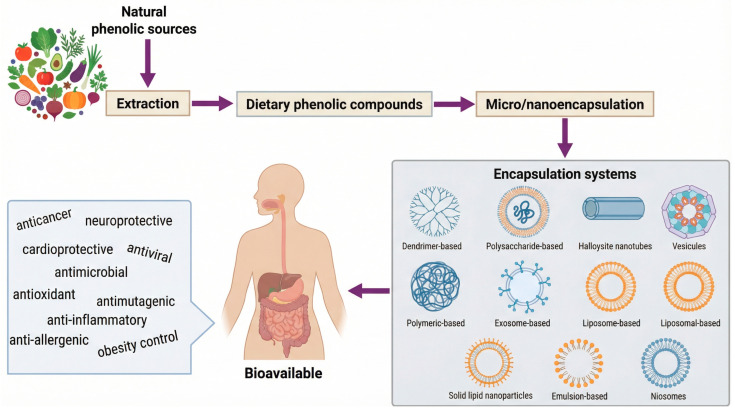
The potential and health benefits of phenolics extracted from natural sources to be applied as pharmaceutical excipients and nanocarriers (adapted from Gonçalves et al. [18]).

Considering human health, several strategies have been studied to encapsulate phenolic compounds to increase their health-promoting properties (Figure 3), and most of these approaches appear to be highly effective (for more information, please read, e.g., [18,95,96,97,98]). Additionally, other efforts have been made to utilize phenolics as excipients and nanocarriers.

In general, excipients and nanocarriers must be chemically stable, non-reactive, inert to the human body, non-toxic, economical, and readily available, with pleasant and effective organoleptic characteristics [13,14]. Furthermore, it is important that they do not interact with medications, packaging, or other excipients [13]. Taking into account the importance of making more effective drugs and/or the fact that most of the conventional excipients and nanocarriers fail in some of these conditions, several efforts have been made to search for better excipients and nanocarriers [99]. Among the alternatives, phenolics seem to be a useful tool in both areas given their chemical structure that confers them extra-functional protection when compared with conventional excipients and nanocarriers (e.g., carboxymethyl starch, chitosan, cyclodextrin, ferritin, mannitol and polyvinylpyrrolidone), including notable antioxidant, anti-inflammatory, antimicrobial, stabilizer and coating of drugs, keeping their effectiveness and safety [100,101].

Anthocyanins are a hot topic of discussion and investigation as new excipients and nanocarriers, particularly in topical/oral formulations [100,102]. In addition, they can also be a powerful tool in drug delivery systems in acidic pH, and act as eco-friendly indicators and sensors, given their capacity to change color in different pH environments [18,103]. Although there is a lack of studies considering this topic, the present part reviewed the latest evidence published in the last decade regarding the use of anthocyanins as excipients and nanocarriers. Since there are a few articles, studies involving other phenolics were also added, as mentioned in Table 2. 

**Table 2:** In vitro and in vivo studies on the use of phenolic compounds (particularly anthocyanins) as excipients and nanocarriers.

Phenolics	System/Nanocarrier	Experimental Model	Main Findings	References
In vitro				
6 mg/mL berberine anthocyanins and 6 mg/mL oligomeric proanthocyanidins	Oligomeric proanthocyanidin excipients	RAW264.7 macrophages and rat articular chondrocyte CP-R09 cells	↓ ROS and inflammatory markers	[100]
Various polyphenols (15 μM tannic acid, 200 μM resveratrol, 200 μM epicatechin gallate, 1000 μM gallic acid and 200 μM procyanidin B2)	Metallic Au@Ag nanoparticles coated with polyphenols	Human HaCaT keratinocytes	↑ Wound healing	[104]
10 mM gallic acid	Silver nanoparticles coated with gallic acid	Two bacteria, *Escherichia coli*, *Staphylococcus aureus*, and one fungus, *Candida albicans*	Antimicrobial activity	[105]
10 mM Gallic acid	Silver nanoparticles coated with gallic acid	Cervical carcinoma HeLa cells	Toxic effects	[105]
38.8 mM Gallic acid	Gallic acid-capped gold nanoparticles	Breast cancer MDA-MB-231 cells	↓ MMP-9 expression by interfering with p300 stabilization and NFκB/c-Jun activation	[106]
42 μM propyl gallate	Synthetic polyphenolic propyl gallate excipients	Liquid pharmaceutical formulations	↑ antioxidant ability of simvastatin and ketoconazole in liquid formulations	[107]
10 mM Gallic acid	Gallic acid carried luminescent ruthenium-modified selenium nanoparticles	Human umbilical vascular endothelial HUVEC and human hepatocellular adenocarcinoma HepG2 cells	↑ Angiogenesis	[108]
10 mM Gallic acid and 10 mM quercetin	Synthesis of bimetallic (Ag-Se) nanoparticles with gallic acid and quercetin	DPPH and ABTS species	↑ Antioxidant ability	[109]
10 mM Gallic acid and 10 mM quercetin	Synthesis of bimetallic (Ag-Se) nanoparticles with gallic acid and quercetin	Dalton lymphoma cells	↑ Anticancer ability	[109]
10 mM Gallic acid and 10 mM quercetin	Synthesis of bimetallic (Ag-Se) nanoparticles with gallic acid and quercetin	Two bacteria, *Escherichia coli* and *Bacillus subtilis*	↑ Antimicrobial ability	[110]
10 mM Gallic acid	Synthesis of Se/Ru nanoparticles with gallic acid	Cervical adenocarcinoma HeLa cells	Toxic effects	[110]
4 mM Caffeic acid	Caffeic acid loaded silver particles	Hepatocellular adenocarcinoma HepG2 cells	Toxic effects, by MMP-2 and MMP-9 expression	[111]
In vivo				
7.1 mg/kg of berberine and oligomeric proanthocyanidins nanoparticles	Oligomeric proanthocyanidin excipients	Intra-articular injection on C57BL/6 mice with induced osteoarthritis	↑ Berberine delivery and efficacy Inhibit synovial inflammation Prevent cartilage degradation	[100]
25% *w*/*v* of polyphenol-modified nanoparticles, including tannic acid, gallic acid, resveratrol, epicatechin gallate and procyanidin B2	Metallic Au@Ag nanoparticles coated with polyphenols	Ear topical application in BALB/c mice	↑ Wound healing	[104]
30 nM Tannic acid	Tannic acid-modified Au@Ag nanoparticles	BALB/c mice	Induced epithelial-tomesenchymal transition-like re-epithelialization	[104]
10 mg/mL Gallic acid	Iron-gallic acid coordination nanoparticles	4T1 Tumor-bearing mice	↓ Cancer cells	[112]

↑: increase, ↓: decrease, MMP: matrix metalloproteinase, ROS: reactive oxygen species.

### 5.1. In Vitro Studies

Huang et al. [100] found that 24 h of treatment with oligomeric proanthocyanidin excipients containing berberine reduced reactive oxygen species, and interleukin (IL)-6, Tumor Necrosis Factor (TNF)-α, and caspase-3 inflammatory markers, and enhancing B-cell lymphoma 2 (BCL-2) anti-apoptotic protein expression in murine macrophages and rat articular chondrocyte cells (RAW264.7 and CP-R092, respectively).

Focusing on other polyphenols, it was already reported that the modification of bimetallic Au@Ag nanoparticles with 15 µM tannic acid, 1000 µM gallic acid, 200 µM resveratrol, 200 µM epicatechin gallate, and 200 µM procyanidin B2 also enhances wound healing in human HaCaT keratinocytes [104]. Additionally, silver nanoparticles coated with gallic acid demonstrated antimicrobial activity against two bacteria, *Escherichia coli* and *Staphylococcus aureus*, and one fungus, *Candida albicans*, with minimum inhibitory concentrations of 6, 30, and 24 g/mL, respectively. The same study also revealed that nanoparticles, at 24 and 30 µg/mL, also showed fewer toxic effects in normal hepatocyte HL-7702 cells when compared with human cervical carcinoma HeLa cells [105]. Chen et al. [106] revealed that gallic acid-capped gold nanoparticles are really effective in inhibiting EGF-induced matrix metalloproteinase-9 (MMP-9) expression through suppression of p300 stabilization and NFκB/c-Jun activation in breast cancer MDA-MB-231 cells.

On the other hand, through in vitro antioxidant assays, Celestino and colleagues [107] revealed that the use of the synthetic polyphenolic propyl gallate as an excipient also revealed the ability to increase the antioxidant ability of simvastatin and ketoconazole in liquid pharmaceutical formulations. Furthermore, gallic acid, carried by luminescent ruthenium-modified selenium nanoparticles at concentrations of 2.5, 5.0, and 10 µg/mL, exhibited angiogenesis in human umbilical vascular endothelial cells (HUVEC) and human hepatocellular adenocarcinoma HepG2 cells [108]. Moreover, the synthesis of bimetallic (Ag-Se) nanoparticles with gallic acid and quercetin increased their anticancer ability in Dalton lymphoma cells, reducing in 80% their viability, as well as their antioxidant activity against DPPH and ABTS species, and antimicrobial potential regarding *Escherichia coli* and *Bacillus subtilis* [109]. The use of gallic acid in the synthesis of Se/Ru nanoparticles makes them more effective in suppressing cervical adenocarcinoma HeLa cells proliferation through the induction of apoptosis and inhibiting their migration and invasion via the inhibition of MMP-2 and MMP-9 proteins [110]. As well as gallic acid, caffeic acid-loaded silver particles showed cytotoxicity against HepG2 cells [111].

### 5.2. In Vivo Studies

More recently, Huang and colleagues [100] reported that the 3-day injection of oligomeric proanthocyanidin excipients for 4 weeks increased the delivery and efficacy of berberine in C57BL/6 mice with induced osteoarthritis by counteracting its hydrophobic properties and limited solubility in water, and thereby, increasing its long-acting release ability. Additionally, immunological techniques have shown potential to inhibit synovial inflammation and prevent cartilage degradation. Altogether, this data contributes to reducing the drug administration frequency and also represents an alternative to increasing its health effects.

Regarding other polyphenols, the ear topical application (once daily for three consecutive days) of Au@Ag nanoparticles modified with 15 µM tannic acid, 200 µM resveratrol, 200 µM epicatechin gallate, 1000 µM gallic acid, and 200 µM procyanidin B2 induced wound healing in BALB/c mice, without leading to local irritation or inflammation [110]. Additionally, tannic acid-modified Au@Ag nanoparticles induced epithelial–tomesenchymal transition-like re-epithelialization, while other polyphenol modifications of Au@Ag nanoparticles acted through proliferation and wound closure [110]. Additionally, iron-gallic acid coordination nanoparticles showed effectiveness in destroying breast cancer cells, after their intravenous injection into 4T1 tumor-bearing mice for 30 days, with no discernible sign of toxic effects [112].

## 6. Conclusions

Anthocyanins appear to be a promising tool as pharmaceutical topical/oral excipients and nanocarriers, particularly due to their antioxidant properties, which may protect drugs against oxidative degradation, and their potential to aid in targeted delivery in acidic environments. Nevertheless, comprehensive in vitro and in vivo studies, as well as clinical trials, are still required, since anthocyanins are highly sensitive to light, pH, temperature, and oxygen, and can readily interact with other phenolic compounds and/or metal ions. Furthermore, the use of purified and standardized anthocyanins is preferable in order to minimize variability in results.

## Data Availability

No new data were created or analyzed in this study. Data sharing is not applicable to this article.
